# Trends and correlation between antibacterial consumption and carbapenem resistance in gram-negative bacteria in a tertiary hospital in China from 2012 to 2019

**DOI:** 10.1186/s12879-021-06140-5

**Published:** 2021-05-17

**Authors:** Chunhong Liang, Xueyan Zhang, Lijuan Zhou, Guangyi Meng, Liqiu Zhong, Pingzhi Peng

**Affiliations:** Department of Pharmacy, Sixth Affiliated Hospital of Guangxi Medical University, Yulin, 537000 Guangxi China

**Keywords:** Antibacterial consumption, Drug resistance, Microbial, Gram-negative bacteria, Carbapenems

## Abstract

**Background:**

To investigate the trends and correlation between antibacterial consumption and carbapenem resistance in Gram-negative bacteria from 2012 to 2019 in a tertiary-care teaching hospital in southern China.

**Methods:**

This retrospective study included data from hospital-wide inpatients collected between January 2012 and December 2019. Data on antibacterial consumption were expressed as defined daily doses (DDDs)/1000 patient-days. Antibacterials were classified according to the Anatomical Therapeutic Chemical (ATC) classification system. The trends in antimicrobial usage and resistance were analyzed by linear regression, while Pearson correlation analysis was used for assessing correlations.

**Results:**

An increasing trend in the annual consumption of tetracyclines, β-lactam/β-lactamase inhibitor (BL/BLI) combinations, and carbapenems was observed (*P* < 0.05). Carbapenem resistance in *Acinetobacter baumannii* (*A. baumannii*) significantly increased (*P* < 0.05) from 18% in 2012 to 60% in 2019. Moreover, significant positive correlations were found between resistance to carbapenems in *A. baumannii* (*P* < 0.05) and *Escherichia coli* (*E. coli*; *P* < 0.05) and consumption of carbapenems, while the resistance rate of *A. baumannii* to carbapenems was positively correlated with cephalosporin/β-lactamase inhibitor (C/BLI) combinations (*P* < 0.01) and tetracyclines usage (*P* < 0.05). We also found that use of quinolones was positively correlated with the resistance rate of *Burkholderia cepacia* (*B. cepacia*) to carbapenems (*P* < 0.05), and increasing uses of carbapenems (*P* < 0.01) and penicillin/β-Lactamase inhibitor (P/BLI) combinations (*P* < 0.01) were significantly correlated with reduced resistance of *Enterobacter cloacae* (*E. cloacae*) to carbapenems.

**Conclusion:**

These results revealed significant correlations between consumption of antibiotics and carbapenem resistance rates in Gram-negative bacteria. Implementing proper management strategies and reducing the unreasonable use of antibacterial drugs may be an effective measure to reduce the spread of carbapenem-resistant Gram-negative bacteria (CRGN), which should be confirmed by further studies.

## Background

Increasing antibacterial resistance and associated infections have become a global health threat [1]. Antibacterial resistance can lead to unresponsiveness to treatment, resulting in persistent illness and increased risk of death [2]. Multidrug-resistant organisms harm not only patients who are infected but also those not infected with multidrug-resistant bacteria. All patients are affected by the lack of appropriate antibiotic regimens due to less efficient antimicrobial agents [3].

Among the many antibiotic-resistant bacteria infections, carbapenem-resistant Gram-negative bacteria (CRGN), which mainly include carbapenem-resistant Enterobacteriaceae (CRE), *Acinetobacter baumannii* (CRAB) and *Pseudomonas aeruginosa* (CRPA), pose a significant threat to humanity in terms of mortality, medical burden, and trends in antimicrobial resistance [4]. The emergence and spread of CRGN are considered to be mainly related to the hydrolysis of carbapenemase, changes in membrane permeability [5], overuse of antimicrobials, and healthcare-associated infections [6]. In a report, the European Union and European Economic Area estimated that the burden of carbapenem-resistant *Klebsiella pneumonia* (CRKP) increased the most (by 6.16 times) among the bacterial species studied in terms of the number of infections and deaths during 2007–2015, followed by carbapenem-resistant *Escherichia coli* (CREC) [7]. In the face of rapidly increasing antimicrobial resistance, the World Health Organization (WHO) ranked CRGN as the most urgent bacteria in 2017 [8].

According to data reported by the China Antibacterial Surveillance Network, the usage rate and defined daily doses (DDDs)/1000 patient-days in inpatients have shown a downward trend from 2011 to 2017 since the rectification of the clinical application of antimicrobials in China in 2011 [9]. However, consumption of carbapenems has increased, with a rise of carbapenem resistance rate in Gram-negative bacteria. Therefore, it was proposed to strengthen the management of carbapenems and tigecycline to reduce carbapenem resistance in China in February 2017. It was shown that reduced antibacterial use is positively associated with improved bacterial resistance without negatively affecting medical quality indexes [10]. Since use of antibiotics can create selective pressure favoring the spread of resistance [11], assessing the association of antibiotic usage with antimicrobial resistance could help local physicians and decision-makers make better use of antibacterials and distribute healthcare funds more effectively, while improving infection control strategies [12]. Therefore, the aim of this study was to describe the changing trend of antibacterial usage and the prevalence of CRGN from 2012 to 2019 in a tertiary hospital and to evaluate the correlation between them.

## Methods

### Study design and setting

The data in this study were collected from local monitoring of carbapenem resistance in Gram-negative bacteria and antibacterial consumption at the Sixth Affiliated Hospital of Guangxi Medical University, which is a comprehensive, tertiary care, university-affiliated, and teaching hospital in the south of China. It is also the largest hospital in Southeast Guangxi, with currently 2400 available beds.

### Bacterial isolates and susceptibility testing

Antibiotic resistance data, which were provided by the hospital’s clinical microbiology laboratory, were extracted from the hospital information system. A total of 37,716 cases with data on Gram-negative bacterial resistance were recorded, including all positive clinical specimens, and 34,489 cases with data on six isolated species, including A*. baumannii, Pseudomonas aeruginosa (P. aeruginosa), Klebsiella pneumonia (K. pneumonia), E. coli, E. cloacae, and B. cepacia*, which were target bacteria to be monitored in this hospital. The resistance rate was reported as the percentage of resistant isolates among all tested isolates. Antimicrobial susceptibility was tested based on the latest performance standards set by the Clinical and Laboratory Standards Institute (CLSI). Tested isolates that had intermediately resistance were eliminated from this analysis. We retained the first isolate of duplicate test results from the same patient during the same in-patient stay. The isolation rate was calculated as the percentage of total gram-negative bacterial isolates.

### Antibacterial consumption

Antibacterial consumption data from 2012 to 2019 were obtained from the hospital information system. According to the Guidelines for Anatomical Therapeutic Chemical (ATC) classification and DDD assignment 23rd edition (2020, [13]), which was developed by the WHO, data on antibacterial consumption were expressed as DDDs/1000 patient-days. Antibacterials were classified according to the ATC classification system.

### Statistical analysis

The changing trends of antimicrobial use and resistance were analyzed by linear regression. Pearson correlation analysis was used for evaluating the relationship between antibiotic consumption and bacterial resistance rates. SPSS 18.0 (SPSS, USA) was used for statistical analysis. A *p*-value < 0.05 was considered statistically significant.

## Results

### Antibacterial consumption

**Fig. 1:**
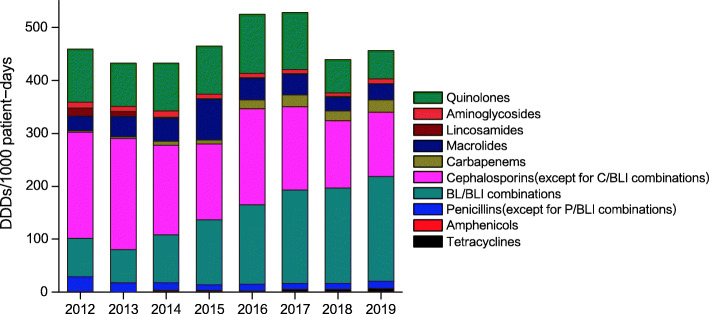
Inpatient antibiotic use for the entire study period. BL/BLI: β-lactam/β-lactamase inhibitor; C/BLI: cephalosporin/β-lactamase inhibitor; P/BLI: penicillin/β-Lactamase inhibitor. Annual consumption of antibiotics was expressed in defined daily doses (DDDs)/1000 patient-days for various antibiotic classes

In this study, consumption of antibiotics throughout the years was first assessed. Figure 1 and Table 1 show the DDDs/1000 patient-days and annual usage trends of antibiotics. During the entire study period, the six top-ranked consumed classes of antimicrobial agents were cephalosporins [except for cephalosporin/β-lactamase inhibitor (C/BLI) combinations], β-lactam/β-lactamase inhibitor (BL/BLI) combinations, quinolones, macrolides, penicillins [except for penicillin/β-Lactamase inhibitor (P/BLI) combinations] and carbapenems. The carbapenem class of antibiotics contained three members in this hospital, including meropenem, imipenem, and biapenem, all of which showed an increasing trend. Among all antibiotics, the most frequently used was piperacillin-tazobactam, followed by cefuroxime and levofloxacin. The annual consumption of several antibiotics significantly decreased during this period, including amphenicols, cephalosporins (except for C/BLI combinations), and lincosamides (*P* < 0.05). Additionally, there was an increasing trend in the annual consumption of tetracyclines, BL/BLI combinations, and carbapenems (*P* < 0.05). No statistically significant variation was observed in the consumption of macrolides, aminoglycosides, and quinolones (*P* > 0.05).

**Table 1:** Trends of annual antibacterial consumption during the entire study period

Antimicrobial class	Annual consumption of antibiotics(DDDs/1000 patient-days)								Linear regression	
	2012	2013	2014	2015	2016	2017	2018	2019	P	Trend
Tetracyclines	0.71	0.06	2.87	4.07	2.49	5.28	5.08	6.78	0.001	Increasing
Amphenicols	1.06	0.91	0.76	0.44	0.49	0.57	0.53	0.45	0.009	Decreasing
Penicillins (except for P/BLI combinations)	26.98	16.46	14.85	10.06	11.85	10.79	12.24	13.84	0.072	No statistically significant difference
BL/BLI combinations	73.09	63.88	90.08	122.26	149.85	176.25	179.49	197.33	0.000	Increasing
P/BLI combinations	57.47	42.74	63.39	89.29	111.45	143.94	145.26	165.17	0.000	Increasing
C/BLI combinations	15.62	21.14	26.69	32.97	38.40	32.31	34.24	32.17	0.020	Increasing
Cephalosporins (except for C/BLI combinations)	200.71	208.67	169.56	170.48	182.50	157.53	126.69	121.53	0.001	Decreasing
First-generation	31.01	23.33	18.97	14.49	13.58	10.72	9.05	8.86	0.000	Decreasing
Second-generation	84.40	99.06	98.36	97.53	105.56	80.33	71.46	74.49	0.135	No statistically significant difference
Third-generation	85.30	86.27	52.23	58.46	63.35	66.47	46.17	38.18	0.013	Decreasing
Carbapenems	3.09	3.97	7.20	11.55	16.34	22.93	19.23	23.84	0.000	Increasing
imipenem	1.72	1.58	2.91	3.76	4.16	4.64	3.47	4.46	0.008	Increasing
meropenem	1.37	2.39	4.29	7.79	12.15	16.71	13.58	16.13	0.000	Increasing
biapenem	0	0	0	0	0.02	1.59	2.19	3.25	0.015	Increasing
Macrolides	26.84	38.66	45.00	45.19	40.44	38.58	26.82	30.11	0.580	No statistically significant difference
Lincosamides	16.09	9.00	0.58	0.63	0.68	0.43	0.22	0.24	0.027	Decreasing
Aminoglycosides	10.52	9.63	11.98	9.08	9.11	8.56	6.94	9.15	0.072	No statistically significant difference
Quinolones	100.35	81.53	90.38	91.47	112.51	108.03	63.12	54.02	0.225	No statistically significant difference

*BL/BLI* β-lactam/β-lactamase inhibitor, *C/BLI* cephalosporin/β-lactamase inhibitor, *P/BLI* penicillin/β-Lactamase inhibitor

Annual consumption of antibiotics was expressed in defined daily doses (DDDs)/1000 patient-days for various antibiotic classes

### Bacterial resistance

Next, bacterial isolates showing carbapenem resistance were assessed.

#### Bacterial isolates

**Fig. 2:**
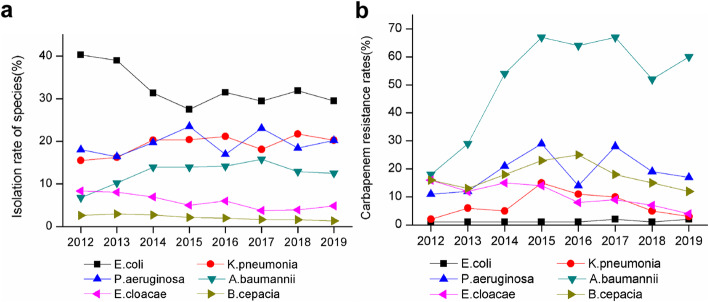
Trends of isolation rate of Gram-negative bacteria and resistance rates of carbapenem-resistant Gram-negative bacteria for the entire study period. **a** Trends of isolation rate of Gram-negative bacteria. **b** Trends of resistance rates of carbapenem-resistant Gram-negative bacteria

Consecutive, non-duplicate bacterial isolates were collected from patients treated at the hospital. Altogether 11,813 *E. coli* isolates, 7412 *K. pneumonia* isolates, 7583 *P. aeruginosa* isolates, 4936 *A. baumannii* isolates, 2007 *E. cloacae* isolates, and 738 *B. cepacia* isolates detected between 2012 and 2019 were included in the analysis. Figure 2a shows the trend of the isolation rate of these species. Isolation rates for *K. pneumonia*, *P. aeruginosa*, and *A. baumannii* showed no statistically significant differences (*P* > 0.05), while *E. coli*, *E. cloacae,* and *B. cepacia* showed a downward trend over recent years (*P* < 0.05).

#### Carbapenem resistance

Figure 2b shows carbapenem resistance trends in Gram-negative bacteria. Carbapenem resistance in *A. baumannii* significantly increased (*P* < 0.05) from 18% in 2012 to 60% in 2019. In contrast, a significant decrease in carbapenem resistance of *E. cloacae* was observed (*P* < 0.01). However, the remaining four Gram-negative isolates had no significant differences in carbapenem resistance (*P* > 0.05).

#### Correlation between antibacterial consumption and carbapenem resistance

**Fig. 3:**
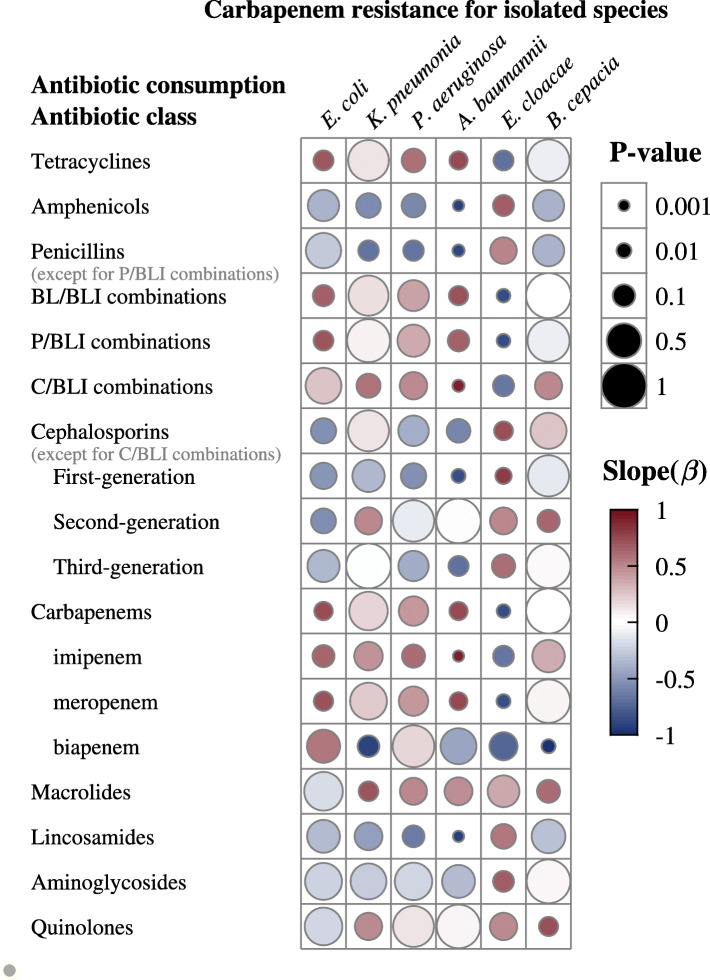
Associations of resistance to carbapenems with antibacterial usage for the entire study period. BL/BLI: β-lactam/β-lactamase inhibitor; C/BLI: cephalosporin/β-lactamase inhibitor; P/BLI: penicillin/β-Lactamase inhibitor. Consumption of antibiotic was expressed in defined daily doses (DDDs)/1000 patient-days

**Fig. 4:**
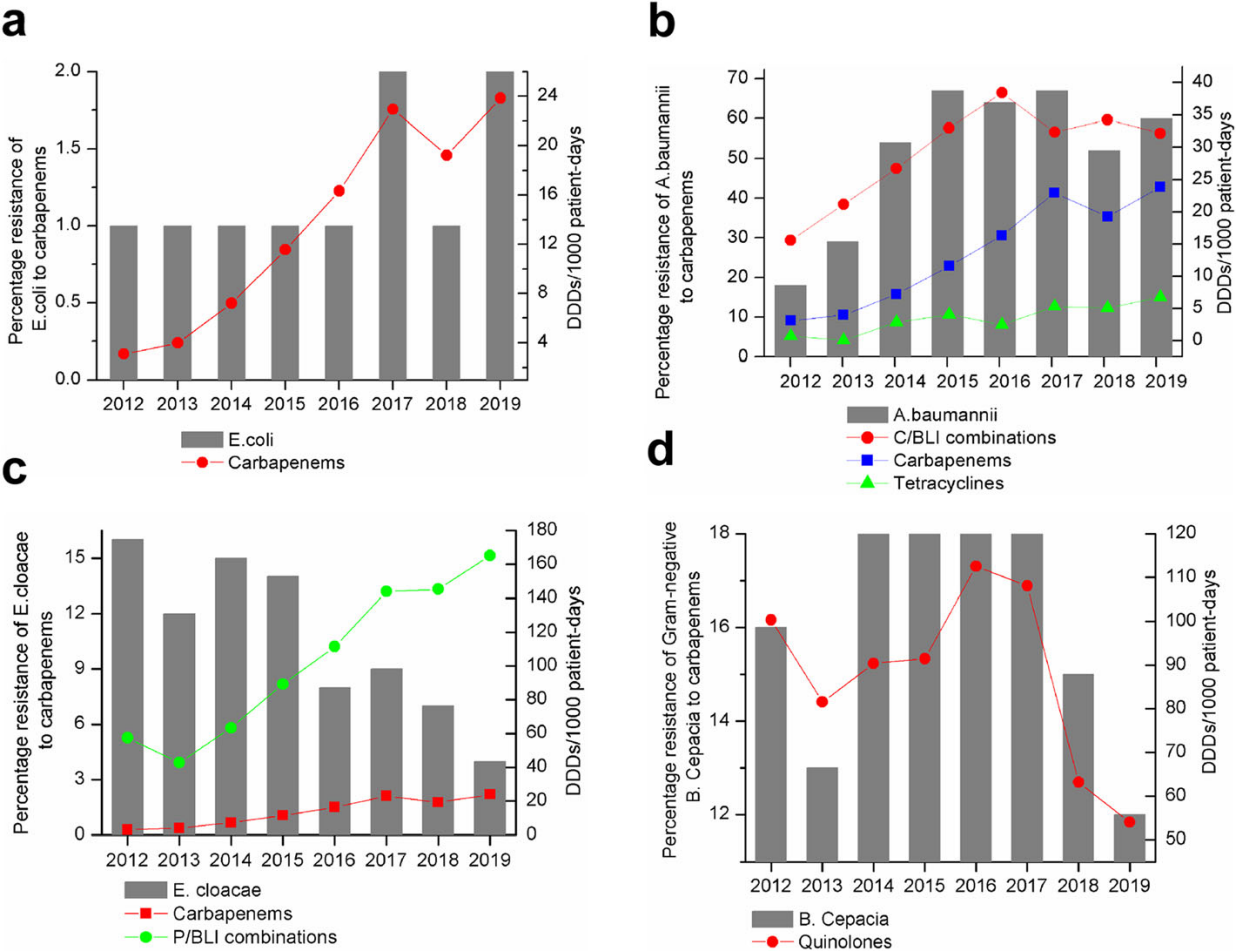
Associations of antibiotic usage and carbapenem resistance rates in *E. coli*, *A. baumannii*, *E. cloacae*, and *B. cepacia* in the entire study period. **a** Correlation between usage of carbapenems and carbapenem resistance in *E. coli*. **b** Associations of usage of tetracyclines, carbapenems, and C/BLI combinations with carbapenem resistance in *A. baumannii*. **c** Associations of usage of carbapenems and P/BLI combinations with carbapenem resistance in *E. cloacae*. **d** Correlation between consumption of quinolones and carbapenem resistance in *B. cepacia*. C/BLI: cephalosporin/β-lactamase inhibitor; P/BLI: penicillin/β-Lactamase inhibitor. Consumption of antibiotic was expressed in defined daily doses (DDDs)/1000 patient-days

**Fig. 5:**
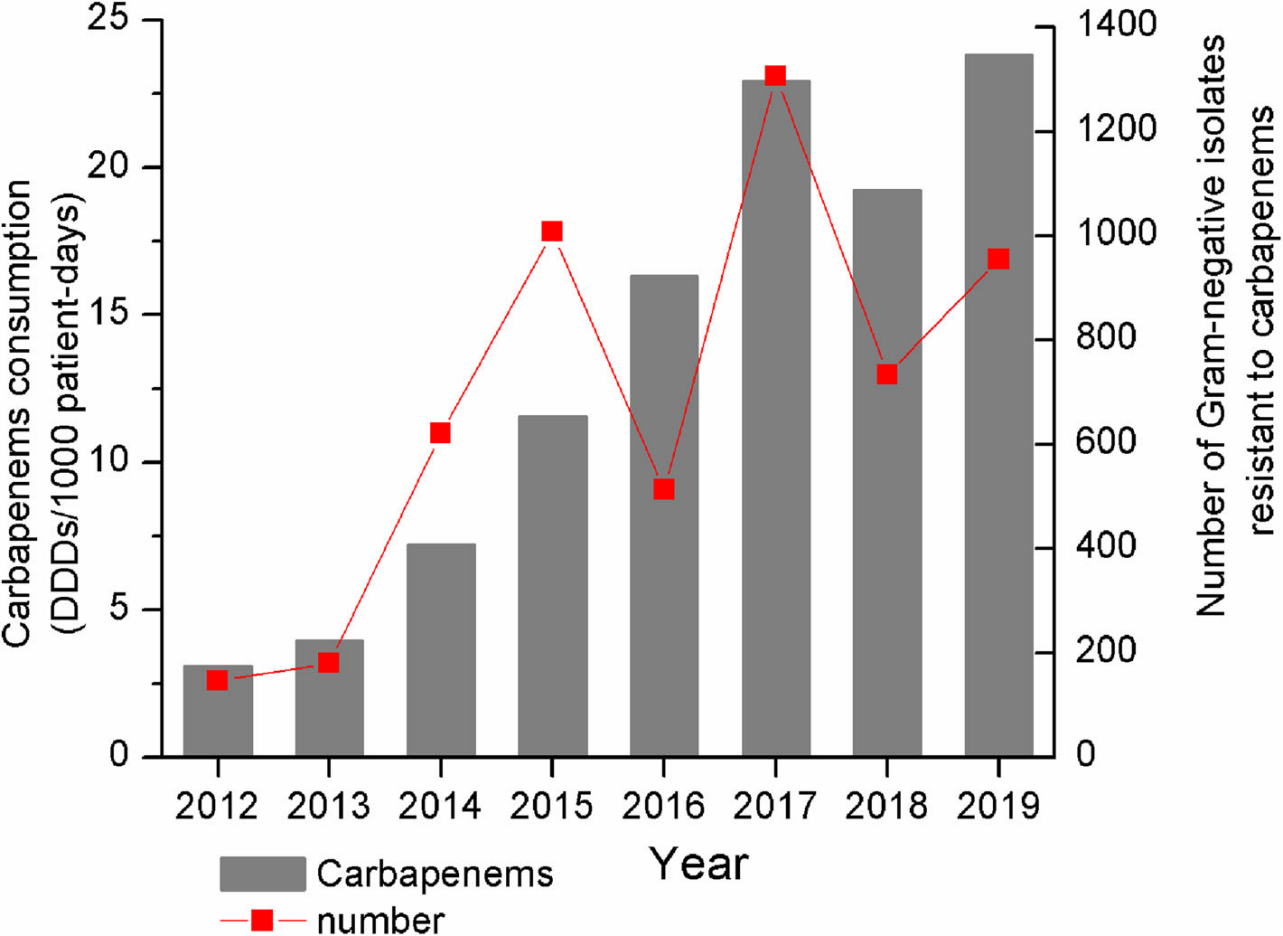
Correlation between carbapenem usage and the number of Gram-negative isolates resistant to carbapenems in the entire study period. Consumption of carbapenems was expressed in defined daily doses (DDDs)/1000 patient-days

In correlation analysis, Figs. 3 and 4 show associations of resistance to carbapenem with antibacterial usage for different classes from 2012 to 2019. Significant positive associations were found of consumption of tetracyclines (*P* < 0.05), BL/BLI combinations (*P* < 0.05), C/BLI combinations (only including cefoperazone-sulbactam) (*P* < 0.01), meropenem (*P* < 0.05), imipenem (*P* < 0.01) and carbapenems (*P* < 0.05) with the rate of CRAB. Conversely, there were significant negative associations of consumption of penicillins (except for P/BLI combinations) (*P* < 0.01), amphenicols (*P* < 0.01), first-generation cephalosporins (*P* < 0.01) and lincosamides (*P* < 0.01) with the rate of CRAB. Moreover, usage of meropenem (*P* < 0.05) and carbapenems (*P* < 0.05) were positively correlated with the rate of CREC. Carbapenem-resistant *E. cloacae* showed significant negative correlations with usage of P/BLI combinations (*P* < 0.01), BL/BLI combinations (*P* < 0.01), meropenem (*P* < 0.01) and carbapenems (*P* < 0.01), but a significant positive correlation with total cephalosporins (except for C/BLI combinations) use (*P* < 0.05). Moreover, carbapenems resistance in *B. cepacia* was positively correlated with quinolones use (*P* < 0.05). Unexpectedly, there was no significant correlation between antibacterial consumption and carbapenem resistance in *P. aeruginosa* and *K. pneumonia (P* > 0.05). Moreover, we found that the rise of carbapenems usage was consistent with an increase in the number of Gram-negative isolates resistant to carbapenems (*P* < 0.05) (Fig. 5).

## Discussion

The present study revealed significant associations of consumption of antibiotics with the rates of carbapenem resistance in Gram-negative bacteria.

As shown above, cephalosporins (except for C/BLI combinations) had the largest consumption variety, despite showing a downward trend. In addition, consumption of BL/BLI combinations showed an increasing trend, and piperacillin-tazobactam was the most frequently used antibiotic combination, corroborating previous findings [14]. It is known that a history of using 3rd generation cephalosporins independently predicts extended-spectrum β-lactamase (ESBL)-producing *K. pneumoniae* or *E. coli* bacteremia [15]. The replacement of 3rd generation cephalosporins by BL/BLI combinations is suitable for reducing the incidence of ESBL-producing Enterobacteriaceae [16–18]. Additionally, our results revealed significantly increased consumption of carbapenems, corroborating global trends [19, 20]. Furthermore, the present findings confirmed that elevated carbapenem usage was associated with increased number of infections resistant to carbapenems. Yet, it is undeniable that overuse of carbapenems presents an important problem, and carbapenems should be used more rationally.

In this study, the isolation rates of *E. coli*, *E. cloacae*, and *B. cepacia* showed a downward trend. As expected, *E. coli* was the most common isolate in 2012–2019, but still highly sensitive to carbapenems. Carbapenem resistance rates in *K. pneumoniae* were significantly lower than the national levels and relatively stable, ranging from 2% in 2012 to 3% in 2019. Previous studies in China revealed CRKP incidence increased from 8.9% in 2005 to 26.3% in 2018 [21, 22]. Surprisingly, the detection rate of CRKP declined for the first time in 2019 [21].

CRAB had the highest rate among all assessed species, rapidly rising between 2012 and 2019, as reported for the whole country [21–23]. Since *A. baumannii* has a resistance rate above 50% to most antibacterial drugs, susceptibility testing should always be performed before its application [24]. Our results showed significant positive associations of resistance to carbapenems in *A. baumannii* and *E. coli* with carbapenem consumption, corroborating a large multicenter study in China [25]. Previous studies have indicated that CRAB rate increase is related to carbapenem exposure [26, 27].

Different from findings by Tan et al [28], which showed no significant association between usage of BL/BLI combinations and CRAB prevalence, *A. baumannii* resistance to carbapenems was positively correlated with cefoperazone-sulbactam usage in the present study. We also observed a positive correlation between tetracycline consumption and *A. baumannii* resistance to carbapenems. The cefoperazone/sulbactam-based combination regimen, which is usually combined with tigecycline, minocycline, carbapenems or aminoglycosides, is the commonest treatment option for carbapenem-resistant and extensively drug-resistant *A. baumannii* infections [29]. Therefore, it is understandable that increased consumption of cefoperazone-sulbactam and tetracyclines is associated with carbapenem resistance in *A. baumannii*.

Unexpectedly, increasing use of carbapenems and P/BLI combinations was significantly correlated with reduced resistance of *E. cloacae* to carbapenems. Nevertheless *E. cloacae* has genomic heterogeneity, and consumption of carbapenems is an independent risk factor for infection of imipenem-heteroresistant *E. cloacae* [30], which may be selected for highly resistant and pathogenic strains. Accordingly, this finding did not provide a strong basis for the selection of antimicrobials to *E. cloacae* infections. Given the lack of related research on the correlation between antibacterial consumption and carbapenem resistance in *E. cloacae*, further research is needed to verify this association. It is difficult to treat infections caused by *B. cepacia*, because of the high level of intrinsic and acquired resistance to many antimicrobial agents. Carbapenems are one of the most reliable antibacterial drugs [31]. Yet, we found that use of quinolones was positively correlated with *B. cepacia* resistance to carbapenems, which prompts more attention to be paid to the use of quinolones to slow down carbapenem resistance in *B. cepacia*.

Because of the urgent need to improve the rational use of antibiotics, a multidisciplinary antibiotic team, comprising infectious disease specialists, microbiologists, clinical pharmacists, and clinicians, was established in 2017 in our hospital. This team ensures appropriate administration of antibiotic therapy for inpatients, pointing out medication errors and offering corrective suggestions. Coincidentally, carbapenem resistance rates in *K. pneumoniae, P. aeruginosa, E. cloacae,* and *B. cepacia* showed a downward trend from 2017 to 2019. Consequently, reducing the unreasonable use of antibacterial drugs may be an effective measure for reducing the spread of CRGN; however, further studies are needed to confirm these observations.

There were some limitations in this study. Firstly, it was conducted only at one tertiary hospital, while carbapenem resistance rates and antibacterial consumption may vary widely across hospitals; therefore, a multicenter study with more relevant data is needed to explore the correlation between antibacterial consumption and CRGN. In addition, due to the retrospective nature of this study, safety could not be examined, and appropriate and inappropriate uses of antibiotics could not be clearly distinguished. Furthermore, the development of carbapenem resistance in Gram-negative infection is associated with various factors, and the selective pressure of antibacterial drugs is only one of them. Multi-factor analysis needs to be further studied.

## Conclusion

The present study demonstrated significant correlations between consumption of antibiotics and the rates of CRGN. Implementing proper management strategies and reducing the unreasonable use of antibacterial drugs may be an effective measure for reducing the spread of CRGN, which needs to be further verified in future studies.

## Data Availability

The datasets used and/or analysed during the current study are available from the corresponding author on reasonable request.

## Abbreviations

*A. baumannii*: *Acinetobacter baumannii*
*E. coli*: *Escherichia coli*
*B. cepacia*: *Burkholderia cepacia*
*E. cloacae*: *Enterobacter cloacae*
*P. aeruginosa*: *Pseudomonas aeruginosa*
*K. pneumonia*: *Klebsiella pneumonia*
CRGN: Carbapenem-resistant Gram-negative bacteria
CRE: Carbapenem-resistant Enterobacteriaceae
CRAB: Carbapenem-resistant *Acinetobacter baumannii*
CRPA: Carbapenem-resistant *Pseudomonas aeruginosa*
CRKP: Carbapenem-resistant *Klebsiella pneumonia*
CREC: Carbapenem-resistant *Escherichia coli*
ESBL: Extended-spectrum β-lactamase
BL/BLI: β-lactam/β-lactamase inhibitor
C/BLI: Cephalosporin/β-lactamase inhibitor
P/BLI: Penicillin/β-Lactamase inhibitor
DDDs: Defined daily doses
ATC: Anatomical Therapeutic Chemical
CLSI: Clinical and Laboratory Standards Institute
MIC: Minimum inhibitory concentration
WHO: World Health Organization
